# Enhancing Associative Learning in Rats With a Computationally Designed Training Protocol

**DOI:** 10.1016/j.bpsgos.2023.07.006

**Published:** 2023-08-01

**Authors:** Xu O. Zhang, Yili Zhang, Claire E. Cho, Douglas S. Engelke, Paul Smolen, John H. Byrne, Fabricio H. Do-Monte

**Affiliations:** Department of Neurobiology and Anatomy, McGovern Medical School, The University of Texas Health Science Center at Houston, Houston, Texas

**Keywords:** Associative memory, Computational model, Fear conditioning, Fear extinction, Long-term potentiation, Spaced learning

## Abstract

**Background:**

Learning requires the activation of protein kinases with distinct temporal dynamics. In *Aplysia*, nonassociative learning can be enhanced by a computationally designed learning protocol with intertrial intervals (ITIs) that maximize the interaction between fast-activated PKA (protein kinase A) and slow-activated ERK (extracellular signal-regulated kinase). Whether a similar strategy can enhance associative learning in mammals is unknown.

**Methods:**

We simulated 1000 training protocols with varying ITIs to predict an optimal protocol based on empirical data for PKA and ERK dynamics in rat hippocampus. Adult male rats received the optimal protocol or control protocols in auditory fear conditioning and fear extinction experiments. Immunohistochemistry was performed to evaluate pCREB (phosphorylated cAMP response element binding)\protein levels in brain regions that have been implicated in fear acquisition.

**Results:**

Rats exposed to the optimal conditioning protocol with irregular ITIs exhibited impaired extinction memory acquisition within the session using a standard footshock intensity, and stronger fear memory retrieval and spontaneous recovery with a weaker footshock intensity, compared with rats that received massed or spaced conditioning protocols with fixed ITIs. Rats exposed to the optimal extinction protocol displayed improved extinction of contextual fear memory and reduced spontaneous recovery compared with rats that received standard extinction protocols. Moreover, the optimal conditioning protocol increased pCREB levels in the dentate gyrus of the dorsal hippocampus, suggesting enhanced induction of long-term potentiation.

**Conclusions:**

These findings demonstrate that a computational model–driven behavioral intervention can enhance associative learning in mammals and may provide insight into strategies to improve cognition in humans.

Long-term memory formation is believed to be mediated by synaptic plasticity including long-term potentiation (LTP) or its invertebrate analogue long-term facilitation (LTF), which requires gene expression and protein synthesis (1, 2, 3, 4, 5). Studies over decades have investigated LTP/LTF and their underlying molecular processes as potential targets to enhance learning or restore memory deficits in laboratory animals. However, interventions using systemic cognitive enhancers or intracerebral pharmacological manipulations (6, 7, 8, 9, 10) have been either based on trial and error approaches in specific model systems or were highly invasive, preventing or hindering translation to humans.

An alternative approach to enhancing learning and memory is to develop computational models to predict optimal learning protocols based on dynamics of intracellular molecular cascades that underlie long-term memory formation and LTP/LTF induction (11, 12, 13). Studies have identified activation of PKA (protein kinase A) (14, 15, 16) and of the MAPK (mitogen-activated protein kinase) isoform ERK (extracellular signal-regulated kinase) (17, 18, 19) as essential cascades for LTF. These 2 pathways converge to phosphorylate transcriptional factors such as CREB (cAMP response element binding protein), which subsequently induce plasticity-related genes during LTF induction (20, 21, 22, 23, 24). These 2 pathways exhibit distinct kinetics of activation (13,16,25), suggesting that the temporal activity patterns, and activation overlap, of these pathways may constitute important targets to enhance associative learning. We previously demonstrated that a computationally designed protocol with irregular intertrial intervals (ITIs), predicted to maximize overlap of PKA and ERK activities, enhances LTF and nonassociative learning, specifically long-term sensitization of the tail-elicited siphon-withdrawal reflex, in *Aplysia* (26).

Substantial similarities between molecular processes of LTF in invertebrates and LTP in mammals make it plausible that strategies used to enhance LTF and nonassociative learning in invertebrates could enhance LTP and associative learning in mammals. PKA activation in rodent hippocampus and amygdala is required for LTP and long-term memory (27,28). Activation of ERK/MAPK cascades and their crosstalk with PKA are required for CREB phosphorylation, induction of plasticity-related genes, and LTP induction (29, 30, 31, 32). Although the dynamics of PKA and ERK activation differ between invertebrates and mammals (33, 34, 35) and may vary across different brain subregions and behavioral protocols in rodents, the chronological order of activation for these 2 intracellular molecules is evolutionarily-conserved across species. In fact, a PKA peak precedes the nuclear translocation of ERK and the subsequent activation of CREB in *Aplysia* (16,25), mice (29), and rats (36, 37, 38), which provides strong support for our model.

Therefore, we tested whether our invertebrate LTF model (26) could be adapted to computationally design an optimal associative learning protocol in mammals. Because the majority of the abovementioned literature on LTP induction has focused on fear conditioning experiments, we used this well-established paradigm to investigate associative learning (39) and used extant empirical data to model PKA and ERK dynamics in rat hippocampus (33, 34, 35), a critical brain region that has been implicated in the formation of associative memories (40, 41, 42). We simulated 1000 different training protocols with varying ITIs to identify an optimal protocol with irregular ITIs that maximizes the overlap of PKA and ERK activities, thereby predicting the formation of stronger memory than training protocols with the same number of stimuli and fixed ITIs. Then, we tested this optimal protocol in auditory fear conditioning and fear extinction paradigms in rats.

## Methods and Materials

See the Supplemental Methods for additional details.

### Animals

The study involved 120 male Long-Evans rats that were aged 3 to 5 months and kept in a 12-hour light/dark cycle. All procedures followed the National Institutes of Health guidelines for animal care and were approved by the Center for Laboratory Animal Medicine and Care of The University of Texas Health Science Center at Houston.

### Model Development

The mathematical model, adapted from Zhang *et al.* (26), describes the activation of the PKA and ERK pathways. Parameters were adjusted based on empirical data (33, 34, 35,43) for the hippocampus. Simulations used fourth-order Runge-Kutta integration for differential equations and were conducted in XPPAUT (44) on Dell Precision T1700 microcomputers.

### Behavioral Tasks

Rats were exposed to fear conditioning in 2 distinct chambers. Three groups—regular conditioning (RC) (8 tone-shock pairings, 270-second intervals), short conditioning (SC) (4 pairings, 270-second intervals), and optimal short conditioning (OSC) (4 pairings, 8-, 8-, and 16-minute intervals)—were used. OSC was compared with spaced short conditioning (SSC) (4 pairings, intervals of 11 minutes 10 seconds) with footshock intensity at 0.5 mA. Extinction involved lever-press training followed by fear conditioning. These rats were divided into 3 groups: regular extinction (RE) (12 tone presentations, 150-second intervals), short extinction (SE) (4 tone presentations, 150-second intervals), and optimal short extinction (OSE) (4 tone presentations, 8-, 8-, and 16-minute intervals).

### Immunohistochemistry

Rat brains were prepared after conditioning for immunohistochemistry to analyze phosphorylated CREB (pCREB)–positive cells. Brains were sectioned and treated with rabbit anti-pCREB serum (1:1000; EMD Millipore 06-519) and biotinylated goat antirabbit IgG antibody (1:200, Vector Labs, BA-1000-1.5), and revealed by ABC kit (1:50, Vector Labs, PK-6100) and DAB-Ni solution (Vector Labs, SK-4100). Images were captured using a Nikon microscope and analyzed with QuPath software (45; https://qupath.github.io) for automatic cell detection and quantification. pCREB-positive cells in specified brain regions were counted, and densities were calculated.

### Quantification and Statistical Analysis

Grubbs’ tests (46) were used to identify outliers in each experiment. Shapiro-Wilk tests were performed to determine the normality of distributions. Equal variance was confirmed through *F* tests and Brown-Forsythe tests. Statistical significance was determined using *t* tests, analysis of variance, or Kruskal-Wallis tests with relevant post hoc comparisons, as appropriate. Sample size was based on previous literature and experience.

## Results

### A Computational Model Based on PKA and ERK Dynamics Identified an Optimal Fear Conditioning Protocol

**Figure 1:**
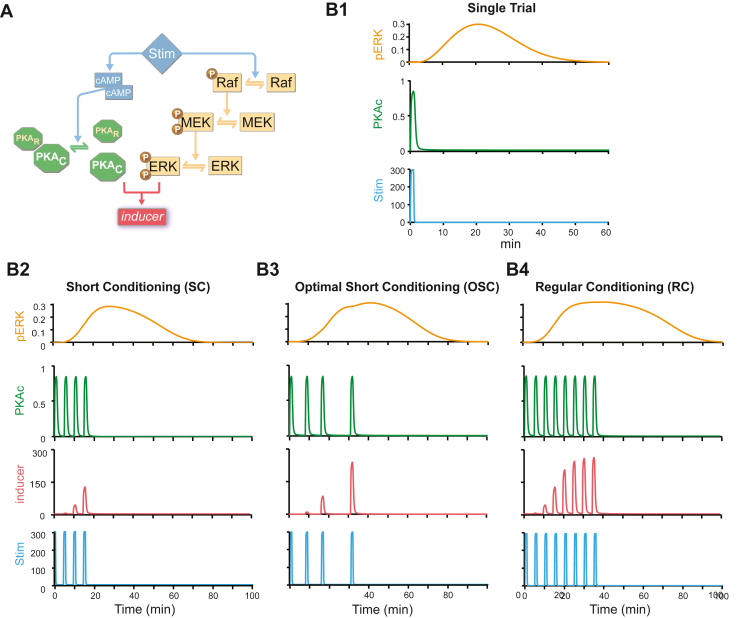
Computational simulations of PKA and ERK pathways predict an optimal protocol for fear conditioning. **(A)** Schematic of the model. Stimulus (μM) rapidly activates PKA via cAMP pathway and activates ERK more slowly via Raf-MEK pathway. The synergistic interaction between PKA and ERK pathways is quantified by a variable inducer, which corresponds to the efficacy of the stimulus in inducing long-term potentiation. ERK kinetics were described by differential equations (see Supplemental Methods), with parameter values reproducing empirical findings that ERK activity reaches peak levels ∼15 to 20 minutes after BDNF treatment or tetanic stimuli in rat hippocampus acute slices (35,43). Equations describing PKA kinetics simulated data showing that PKA is transiently activated within 2 minutes after long-term potentiation induction in slices from rat hippocampus or within 5 minutes in vivo after spatial discrimination task training (33,34). **(B1)** Simulated time courses of activated ERK (pERK, μM) and activated PKA (PKAc, μM) in response to one trial of Stim. **(B2)** Simulated time courses of pERK, PKAc, and inducer in response to a 4-trial protocol with regular intertrial intervals of 4.5 minutes (SC). **(B3)** Simulated time courses of pERK, PKAc, and inducer (nM) in response to a 4-trial protocol with computationally designed intervals (OSC). **(B4)** Simulated time courses of pERK, PKAc, and inducer (nM) in response to an 8-trial protocol with regular ITIs of 4.5 minutes (RC). BDNF, brain-derived neurotrophic factor; cAMP, cyclic adenosine monophosphate; ERK, extracellular signal-regulated kinase; MEK, mitogen-activated protein kinase; OSC, optimal short conditioning; pERK, phosphorylated ERK; PKA, protein kinase A; PKAc, protein kinase A catalytic subunit; PKAr, protein kinase A regulatory subunit; RC, regular conditioning; SC, short conditioning; Stim, stimulus.

The model in Zhang *et al.* (26) was adapted to simulate PKA and ERK dynamics in rat hippocampus during LTP induction (Figure 1A). To the best of our knowledge, the hippocampus is the only rat brain region in which ERK dynamics have been measured with a temporal resolution of minutes following a stimulus. We simulated fear conditioning, in which the pairing of a conditioned stimulus (CS) with an unconditioned stimulus (US) is represented by Stim. The conditioned responses are assumed to be proportional to the peak value of inducer. A single CS-US pairing produced little overlap between PKA and ERK pathways (Figure 1B1). Then, we simulated 1000 fear conditioning protocols, with 4 trials and both fixed and irregular ITIs. These simulations included an SC protocol with 4 trials and a fixed ITI of 270 seconds. As a reference control, we also simulated an RC protocol with 8 trials and the same ITI of 270 seconds. These protocols resemble previous protocols used for short and regular fear conditioning in rats, respectively (47,48). Simulations identified an optimal conditioning protocol with 4 trials and irregular ITIs of 8, 8, and 16 minutes (Figure 1B2–B4), termed OSC. PKA activity induced by the last trial of OSC had a much larger overlap with the phosphorylated ERK curve than did PKA induced by SC. Therefore, OSC induced a much higher peak level of inducer (red). Therefore, we predicted that OSC would produce stronger long-term memory in rats than standard SC.

### The OSC Protocol Increases Fear Memory Compared With the SSC Protocol

**Figure 2:**
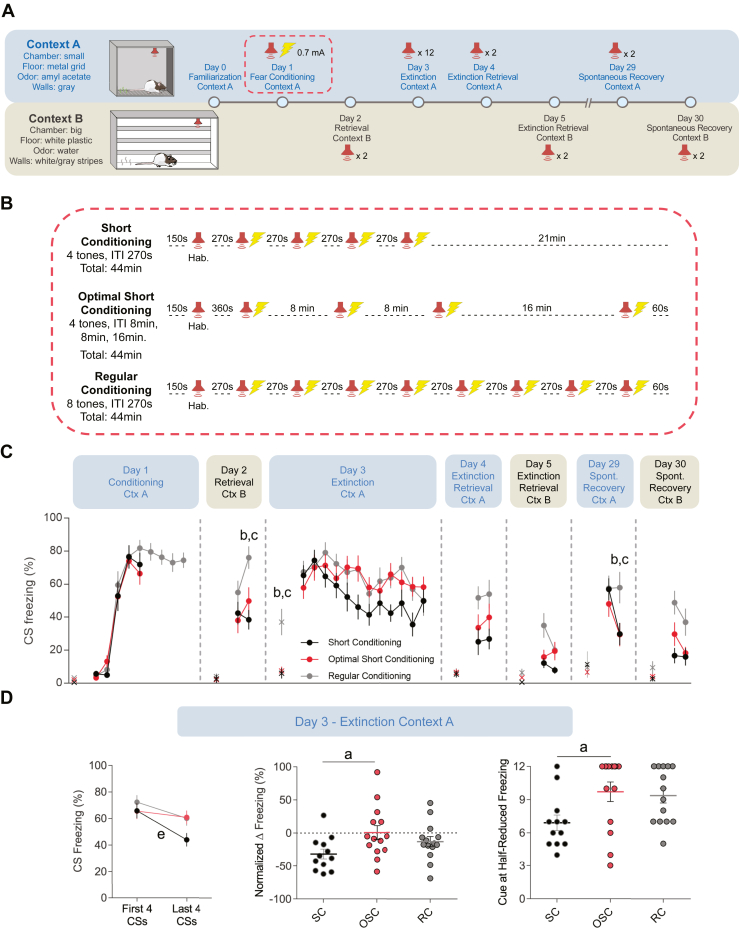
Computationally designed protocol partially enhanced fear conditioning in rats. **(A)** Schematics of the enhanced fear conditioning experimental procedures. (Top panel) Tests that were conducted in Ctx A. (Bottom panel) Tests that were conducted in Ctx B. **(B)** Schematics of the fear conditioning protocols for SC (*n* = 12), OSC (*n* = 14), and RC (*n* = 14) groups. Following a habituation tone, rats received multiple trials of a CS (3 kHz tone, 30 seconds) that coterminated with an unconditioned stimulus (footshock, 0.7 mA, 0.5 seconds). **(C)** Freezing levels during CS presentations of each group across the experiment. Two-way repeated measures analysis of variance for each day followed by Holm-Sidak’s post hoc test. Letters a, b, and c indicate pairwise post hoc tests with *p* < .05: a, OSC vs. SC; b, OSC vs. RC; c, SC vs. RC. × denotes the pretone freezing levels. **(D)** The OSC group is resistant to extinction while the SC group shows significantly more extinction. (Left panel) Freezing levels during the first 4 and last 4 CS presentations. Paired Student’s *t* test. Letter e indicates test with *p* < .05: SC, last 4 CS vs. first 4 CS. (Center panel) Normalized change of the freezing level during extinction, as indicated by the difference in the freezing levels between the last 4 and first 4 CS presentations as a percentage of the freezing level of the first 4 CS presentations ([last 4 CS − first 4 CS]/first 4 CS). One-way analysis of variance followed by Tukey’s post hoc test. Letter a indicates pairwise test with *p* < .05: OSC vs. SC. (Right panel) The number of cues required to reach to a 50% reduction of the original freezing level (average of freezing level during the first 2 cues). Kruskal-Wallis test followed by Dunn’s multiple comparisons test. Letter a indicates pairwise test with *p* < .05: OSC vs. SC. Data are shown here and in the next illustrations as mean ± SEM. CS, conditioned stimulus; Ctx, context; Hab., habituation; ITI, intertrial interval; OSC, optimal short conditioning; RC, regular conditioning; SC, short conditioning; spont., spontaneous.

Because the hippocampus is required for context encoding during auditory fear conditioning (49,50), we used 2 different chambers (context A and context B) to assess the contribution of the context to the auditory fear memory (Figure 2A). Our pilot experiments and a series of recent fear conditioning studies have shown that female rats exhibit higher active defensive responses (i.e., darting) and lower passive defensive responses (i.e., freezing) than male rats (51, 52, 53, 54). Therefore, we performed our experiments in males, which exhibit enhanced freezing behavior, the main index of fear memory adopted in our study. On day 0, rats were familiarized with context A for 20 minutes. They were preassigned to SC, OSC, or RC groups so that baseline freezing and locomotion were similar among groups (Table 1). On day 1, rats were placed into context A and exposed to one nonreinforced habituation tone followed by the SC, OSC, or RC protocols (Figure 2B). All groups reached high levels of freezing (Figure 2C) and reduced locomotion at the end of the fear acquisition session.

**Table 1:** Results of Statistical Analyses

Experiment Day	Description	Statistical Test	Omnibus Test	Omnibus *p* Value	Post Hoc Test	Post Hoc Comparisons	Post Hoc *p* Value
Figure 2							

Day 0	Baseline freezing	One-way ANOVA	*F*_2,37_ = 0.018	.982	NA	NA	NA
	Baseline average speed	One-way ANOVA	*F*_2,37_ = 0.062	.939	NA	NA	NA
	Baseline maximum speed	One-way ANOVA	*F*_2,37_ = 0.044	.956	NA	NA	NA
Day 2	Pretone freezing	Kruskal-Wallis	*H*_2_ = 0.887	.143	NA	NA	NA
	Second CS freezing	Two-way RM ANOVA	*F*_2,37_ = 3.751	.033	Holm-Sidak	OSC vs. SC	.282
						OSC vs. RC	.022
						SC vs. RC	.002
	Average speed	One-way ANOVA	*F*_2,37_ = 10.87	<.001	Tukey	OSC vs. SC	.024
						OSC vs. RC	.131
						SC vs. RC	<.001
Day 3	Pretone freezing	Kruskal-Wallis	*H*_2_ = 13.93	<.001	Dunn	OSC vs. SC	>.999
						OSC vs. RC	.020
						SC vs. RC	.001
	First 2 CS freezing	Two-way RM ANOVA	*F*_22,407_ = 1.169	.271	NA	NA	NA
	First 4 vs. last 4 CS freezing	Paired Student’s *t* test	OSC: *t*_13_ = 0.869	.400	NA	NA	NA
			SC: *t*_11_ = 4.191	.001			
			RC: *t*_13_ = 2.153	.051			
	Relative change in freezing	One-way ANOVA	*F*_2,37_ = 3.315	.047	Tukey	OSC vs. SC	.037
						OSC vs. RC	.487
						SC vs. RC	.320
	Cue at half-reduced freezing	Kruskal-Wallis	*H*_2_ = 7.08	.029	Dunn	OSC vs. SC	.039
						OSC vs. RC	>.099
						SC vs. RC	.095
Day 4	Extinction retrieval CTX A	Two-way RM ANOVA	*F*_2,37_ = 0.097	.907	NA	NA	NA
	Average CS freezing	One-way ANOVA	*F*_2,37_ = 3.944	.028	Tukey	OSC vs. SC	.508
						OSC vs. RC	.508
						SC vs. RC	.023
Day 5	Extinction retrieval CTX B	Two-way RM ANOVA	*F*_2,37_ = 2.984	.063	NA	NA	NA
	Average CS freezing	One-way ANOVA	*F*_2,37_ = 4.067	.025	Tukey	OSC vs. SC	.437
						OSC vs. RC	.236
						SC vs. RC	.020
Day 29	Spontaneous recovery	Two-way RM ANOVA	*F*_2,37_ = 3.259	.049	Holm-Sidak	OSC vs. SC	.976
						OSC vs. RC	.030
						SC vs. RC	.030
Day 30	Spontaneous recovery	Two-way RM ANOVA	*F*_2,37_ = 0.774	.469	NA	NA	NA
Figure 4							

Day 0	Baseline freezing	Unpaired Student’s *t* test	*t*_16_ = 0.988	.337	NA	NA	NA
	Baseline average speed	Unpaired Student’s *t* test	*t*_16_ = 0.419	.680	NA	NA	NA
	Baseline maximum speed	Unpaired Student’s *t* test	*t*_16_ = 0.225	.825	NA	NA	NA
Day 1	Freezing 2nd CS-US pairing	Two-way RM ANOVA	*F*_4,64_ = 1.550	.339	Holm-Sidak planned comparison	OSC vs. SSC	.040
	Freezing 3rd CS-US pairing	Two-way RM ANOVA	*F*_4,64_ = 1.550	.339	Holm-Sidak planned comparison	OSC vs. SSC	.019
Day 3	First 2 CS freezing	Welch’s *t* test	*t*_12.43_ = 2.227	.045	NA	NA	NA
	Freezing end of session (tone 12)	Two-way RM ANOVA	*F*_11,176_ = 2.997	.001	Holm-Sidak	OSC vs. SSC	.987
Day 4	Extinction retrieval CTX A	Two-way RM ANOVA	*F*_1,16_ = 0.349	.563	NA	NA	NA
Day 5	Extinction retrieval CTX B	Two-way RM ANOVA	*F*_1,16_ = 1.130	.304	NA	NA	NA
Day 29	Freezing first CS presentation	Two-way RM ANOVA	*F*_1,16_ = 1.809	.197	Holm-Sidak planned comparison	OSC vs. SSC	.037
Figure 5							

Day 0	Baseline freezing	One-way ANOVA	*F*_2,12_ = 0.970	.407	NA	NA	NA
	Baseline average speed	One-way ANOVA	*F*_2,12_ = 3.706	.056	NA	NA	NA
	Baseline maximum speed	One-way ANOVA	*F*_2,12_ = 3.839	.051	NA	NA	NA
Day 1	Freezing second CS-US pairing	Two-way RM ANOVA	*F*_8,48_ = 1.976	.070	Holm-Sidak planned comparison	OSC vs. SSC	.144
						SSC vs. NS	.029
						OSC vs. NS	.001
	Freezing 3rd CS-US pairing	Two-way RM ANOVA	*F*_8,48_ = 1.976	.070	Holm-Sidak planned comparison	OSC vs. SSC	.312
						SSC vs. NS	.002
						OSC vs. NS	<.001
	Freezing fourth CS-US pairing	Two-way RM ANOVA	*F*_8,48_ = 1.976	.070	Holm-Sidak planned comparison	OSC vs. SSC	.020
						SSC vs. NS	.037
						OSC vs. NS	<.001
	Lateral amygdala IHC	Two-way RM ANOVA	*F*_4,24_ = 2.844	.046	Holm-Sidak	OSC vs. SSC	.838
						SSC vs. NS	<.001
						OSC vs. NS	<.0001
	Basal amygdala IHC	Two-way RM ANOVA	*F*_4,24_ = 2.844	.046	Holm-Sidak	OSC vs. SSC	.420
						SSC vs. NS	.085
						OSC vs. NS	.028
	Central amygdala IHC	Two-way RM ANOVA	*F*_4,24_ = 2.844	.046	Holm-Sidak	OSC vs. SSC	.402
						SSC vs. NS	.010
						OSC vs. NS	.059
	PVT IHC	One-way ANOVA	*F*_2,12_ = 16	<.001	Tukey	OSC vs. SSC	.681
						SSC vs. NS	<.001
						OSC vs. NS	.002
	Hippocampus subfields IHC	Two-way RM ANOVA	*F*_3,36_ = 55.99	<.001	Holm-Sidak	CA1 vs. DG	<.001
						CA2 vs. DG	<.001
						CA3 vs. DG	<.001
	CA1 IHC	Two-way RM ANOVA	*F*_6,36_ = 2.487	.041	Holm-Sidak	OSC vs. SSC	.860
						SSC vs. NS	.416
						OSC vs. NS	.416
	CA2 IHC	Two-way RM ANOVA	*F*_6,36_ = 2.487	.041	Holm-Sidak	OSC vs. SSC	.811
						SSC vs. NS	.153
						OSC vs. NS	.187
	CA3 IHC	Two-way RM ANOVA	*F*_6,36_ = 2.487	.041	Holm-Sidak	OSC vs. SSC	.968
						SSC vs. NS	.465
						OSC vs. NS	.465
	DG IHC	Two-way RM ANOVA	*F*_6,36_ = 2.487	.041	Holm-Sidak	OSC vs. SSC	.049
						SSC vs. NS	.059
						OSC vs. NS	<.001
Figure 7							
Day 8	Fear conditioning freezing and lever presses	One-way ANOVA	*F*_14,231_ = 0.404	.973	NA	NA	NA
	Freezing levels	Kruskal-Wallis	*H*_2_ = 0.188	.910	NA	NA	NA
	Average speed	One-way ANOVA	*F*_2,33_ = 0.042	.958	NA	NA	NA
	Lever press rate	One-way ANOVA	*F*_2,33_ = 0.480	.622	NA	NA	NA
Day 9	CS lever press rate beginning to end	Paired Student’s *t* test	SE: *t*_10_ = 1.000	.341	NA	NA	NA
			OSE: *t*_12_ = 1.237	.239			
			RE: *t*_11_ = 2.837	.016			
	Pre-CS lever press rate beginning to end	Paired Student’s *t* test	SE: *t*_10_ = 1.111	.293	NA	NA	NA
			OSE: *t*_12_ = 3.773	.003			
			RE: *t*_11_ = 0.994	.341			
Day 10	CS freezing	Two-way RM ANOVA	*F*_2,33_ = 0.207	.814	NA	NA	NA
	CS lever press rate	Two-way RM ANOVA	*F*_2,33_ = 0.069	.933	NA	NA	NA
Day 35	CS freezing	Two-way RM ANOVA	*F*_2,33_ = 0.535	.591	NA	NA	NA
	CS lever press rate	Two-way RM ANOVA	*F*_2,33_ = 1.085	.350	NA	NA	NA
	Pre-CS lever press rate (tone 1)	Two-way RM ANOVA	*F*_2,33_ = 2.897	.069	Holm-Sidak planned comparison	OSE vs. SE	.005
						OSE vs. RE	.019
						SE vs. RE	.516
	Average pre-CS lever press rate	Kruskal-Wallis	*H*_2_ = 6.414	.041	Dunn	OSE vs. SE	.035
						OSE vs. RE	.531
						SE vs. RE	.709

ANOVA, analysis of variance; CS, conditioned stimulus; CTX, context; DG, dentate gyrus; IHC, immunohistochemistry; NA, not applicable; OSC, optimal short conditioning; OSE, optimal short extinction; PVT, paraventricular nucleus of the thalamus; RC, regular conditioning; RE, regular extinction; RM, repeated measures; SC, short conditioning; SE, short extinction.

On day 2, rats were placed in context B and given 2 CS presentations to test the retrieval of tone-associated fear memory in a novel context. Compared with the RC group, both the SC and the OSC group exhibited less freezing during the second CS presentation (Figure 2C). However, both RC and OSC groups showed reduced average speed during the retrieval test compared with the SC group, suggesting generalized fear responses in a novel context (Table 1).

On day 3, in context A, rats underwent a retrieval and extinction training session with 12 CS presentations. Only the RC group showed a significantly higher pretone freezing level, suggesting a robust contextual fear memory (Table 1). However, the 3 groups exhibited the same levels of freezing during the first 2 CS presentations, suggesting that tone-evoked fear retrieval was comparable among the groups (Figure 2C). Nevertheless, a within-session extinction analysis comparing the first 4 CS with the last 4 CS revealed impaired extinction learning in the OSC group compared with the SC group, with the OSC group displaying smaller reduction of freezing levels from the first to the last CS presentation (Figure 2D). Despite this extinction impairment, no significant differences were found between the OSC and SC groups during extinction retrieval tests performed on days 4 and 5 or the spontaneous recovery tests performed on days 29 and 30 (Figure 2C). The RC group showed higher averaged CS freezing than the SC group during both extinction retrieval tests (Table 1).

In summary, the OSC protocol demonstrated modest enhancement of fear acquisition, as indicated by reduced locomotion and stronger, more resistant CS-evoked fear memory during within-session extinction. However, this enhancement did not persist during extinction retrieval and spontaneous recovery tests. These results suggest that OSC produces a more robust initial fear memory but without a lasting effect on extinction memory.

### The OSC Protocol Induces Stronger Fear Memory Than an SSC Protocol

**Figure 3:**
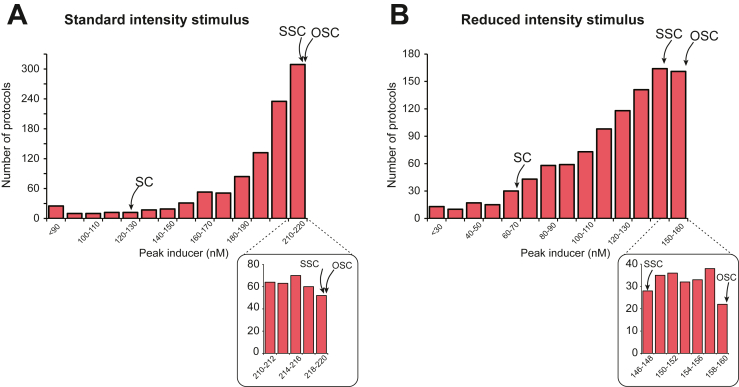
Histogram of peak levels of inducer from 1000 protocols. **(A)** Standard-intensity stimulus (= 300 μM). The range of peak levels of inducer (0–220 nM) was subdivided into 14 bins, and the number of simulations that produced a peak concentration of inducer in each subdivision was plotted. The arrows indicate which bins contained the peak concentrations produced by the SC, SSC, and OSC protocols. **(B)** Reduced-intensity stimulus (= 200 μM). The range of peak levels of inducer (0–160 nM) was subdivided into 14 bins, and the number of simulations that produced a peak concentration of inducer in each subdivision was plotted. Insets below the main plots illustrate in detail the difference in peak inducer for SSC vs. OSC, which is negligible in **(A)**. OSC, optimal short conditioning; SC, short conditioning; SSC, spaced short conditioning.

**Figure 4:**
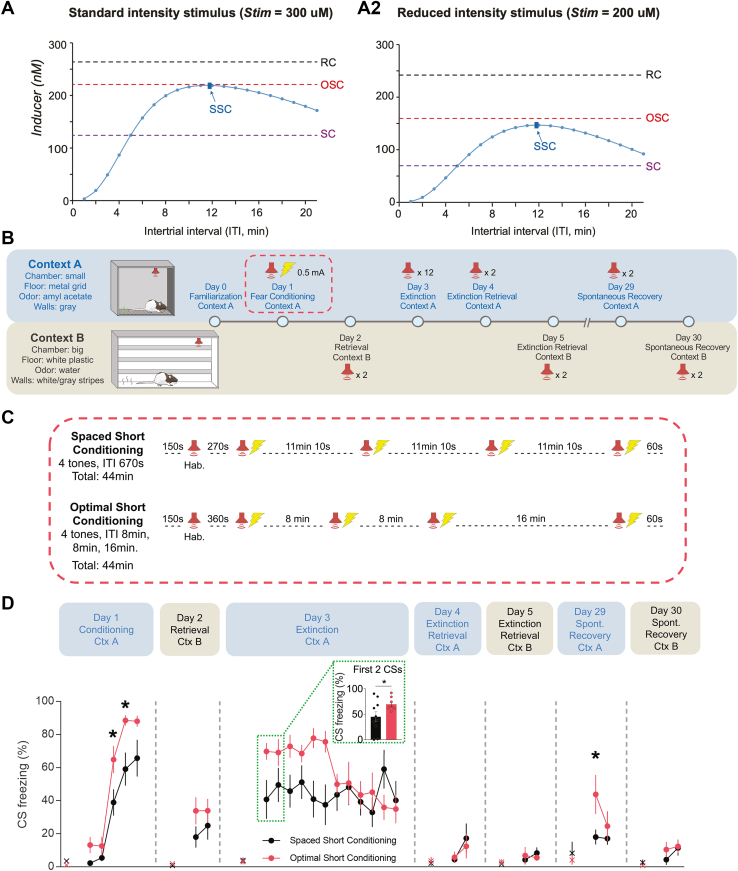
OSC protocol induced stronger fear memory than SSC protocol in rats. **(A)** Inducer peak levels from the RC, SC, and OSC protocols compared with peak levels from SSC protocols of 4 trials with regular ITIs varying from 1 to 21 minutes, using standard-intensity stimulus **(A1)** or reduced-intensity stimulus **(A2)**. The peak inducer values of RC, SC, and OSC are labeled by dashed lines (black: RC; red: OSC; purple: SC). The blue curve gives peak inducer values for the protocols with regular ITIs, and the curve peaks at the dark blue dot and arrow represent the SSC protocol with equal ITIs of 11 minutes 10 seconds. **(B)** Schematics of the fear conditioning procedures. (Top panel) Tests that were conducted in Ctx A. (Bottom panel) Tests that were conducted in Ctx B. **(C)** Schematics of the fear conditioning protocols for SSC (*n* = 10, ITI = 11 minutes 10 seconds) and OSC (*n* = 8) groups. Following a habituation tone, rats received 4 trials of a CS (3 kHz tone, 30 seconds) that coterminated with an unconditioned stimulus (footshock, 0.5 mA, 0.5 seconds). **(D)** Freezing levels during CS presentations of each group across the experiment; × denotes the pretone freezing levels. Two-way repeated measures analysis of variance for each day followed by Holm-Sidak’s pairwise post hoc test, ∗*p* < .05. Inset: the average freezing level during the first 2 CS presentations; Welch’s *t* test, ∗*p* < .05. CS, conditioned stimulus; Ctx, context; Hab., habituation; ITI, intertrial interval; OSC, optimal short conditioning; RC, regular conditioning; SC, short conditioning; spont., spontaneous; SSC, spaced short conditioning; Stim, stimulus.

Spaced learning protocols result in stronger memories than massed learning protocols in both humans (55,56) and rodents [(57, 58, 59), but also see (60)], raising the possibility that the augmented fear memory observed in the OSC group could simply be attributed to a trial-spacing effect. Our simulations predicted a broad range of effective simulated 4-trial protocols (Figure 3). One protocol with relatively long, fixed ITIs of 11 minutes 10 seconds produced a nearly identical peak level of inducer as the OSC protocol and a higher peak level of inducer than other protocols with fixed ITIs from 1 to 21 minutes (Figures 3A and 4A1). We termed this protocol the SSC protocol. Further simulations with reduced stimulus intensity showed that the OSC protocol still produced the greatest peak level of inducer and was more effective than SSC with these weaker stimuli (Figures 3B and 4A2). Therefore, we predicted that the irregular intervals of OSC might produce more effective conditioning than regularly spaced SSC.

To test this prediction, we empirically compared the SSC and OSC protocols using a reduced shock intensity (0.5 mA instead of the 0.7 mA used in Figure 2) (Figure 4B, C). Rats that were exposed to OSC showed higher CS freezing during the second and third CS-US pairing of the fear acquisition session (Figure 4D, day 1). In addition, fear memory retrieval was increased when rats in the OSC group were returned to context A for an extinction training session on day 3, as indicated by higher freezing during the first 2 CS presentations than the SSC group (Figure 4D, Inset). The OSC group and the SSC group exhibited the same levels of freezing by the end of the extinction training session (Figure 4D, day 3), as well as during the extinction retrieval tests that were performed in context A (Figure 4D, day 4) or context B (Figure 4D, day 5). However, the OSC group showed greater spontaneous recovery of fear memory in context A approximately 3 weeks later, as indicated by higher freezing during the first CS presentation than SSC-trained rats (Figure 4D, day 29). In summary, these data suggest that the enhancement in fear memory acquisition that we observed with our OSC protocol cannot simply be attributed to a trial-spacing effect or differences in the delay to remove the animals from the chamber, but rather it is associated with the maximized overlap between PKA and ERK signaling.

### The OSC Protocol Induces Greater Levels of pCREB in Dentate Gyrus Than an SSC Protocol

**Figure 5:**
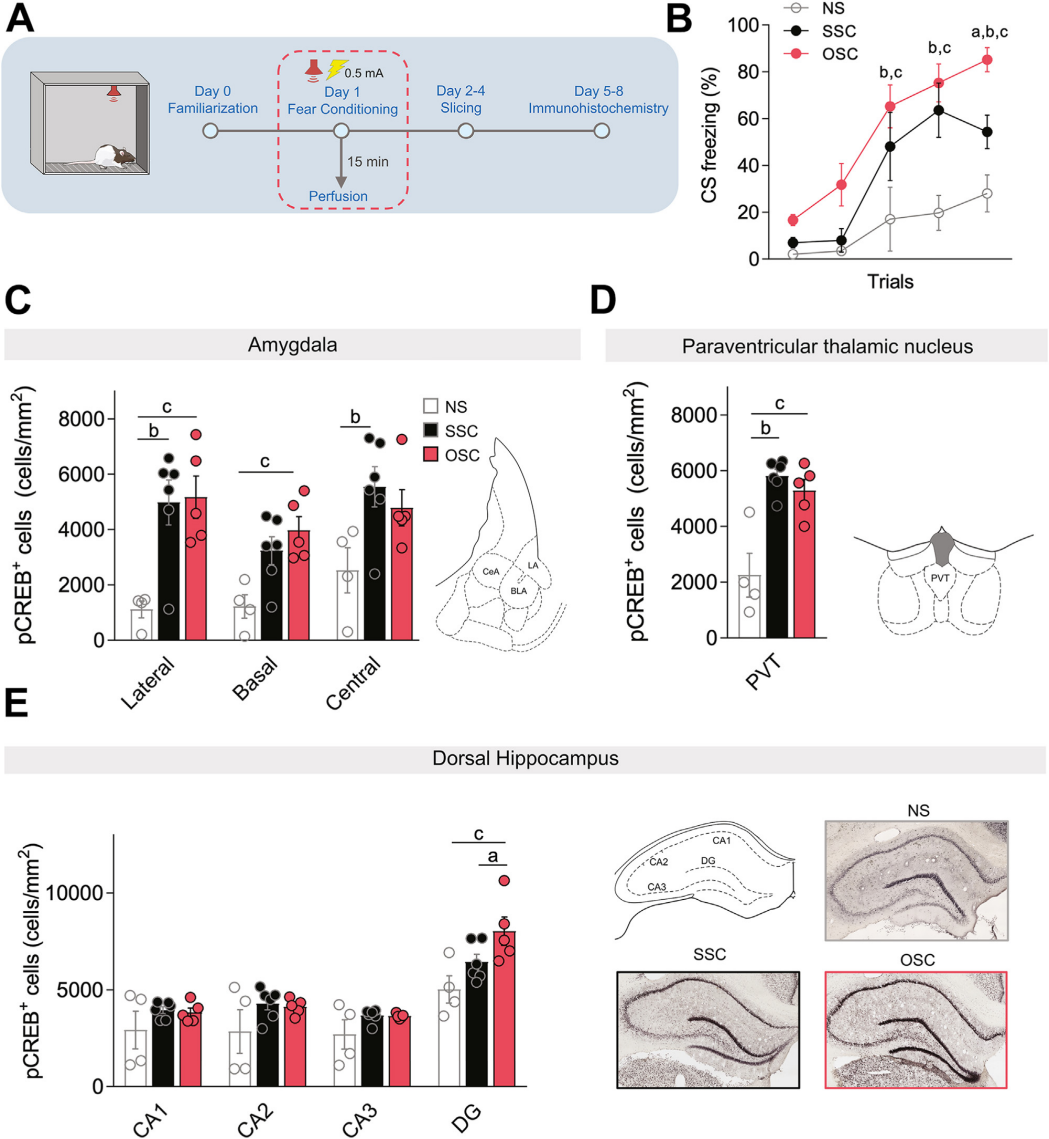
Immunohistochemistry quantification of pCREB in rats exposed to OSC and SSC protocols. **(A)** Schematic of the immunohistochemistry procedure. **(B)** Freezing levels during CS presentation for NS (*n* = 4), SSC (*n* = 6), and OSC (*n* = 5) groups during fear conditioning; two-way repeated measures ANOVA followed by Holm-Sidak’s post hoc test. Letters a, b, and c indicate pairwise post hoc tests with *p* < .05: a, OSC vs. SSC; b, SSC vs. NS; c, OSC vs. NS. **(C)** Average pCREB density in the LA, BLA, and CeA for NS, SSC, and OSC groups; two-way repeated measures ANOVA followed by Holm-Sidak’s post hoc test. Letters b and c indicate pairwise post hoc tests with *p* < .05: b, SSC vs. NS; c, OSC vs. NS. **(D)** Average pCREB density in the PVT for NS, SSC, and OSC groups; one-way ANOVA followed by Tukey’s post hoc test. Letters b and c indicate pairwise post hoc tests with *p* < .05: b, SSC vs. NS; c, OSC vs. NS. **(E)** (Left panel) Average pCREB density across hippocampal subfields for NS, SSC, and OSC groups; two-way repeated measures ANOVA followed by Holm-Sidak’s post hoc test. Letters a and c indicate pairwise post hoc tests with *p* < .05: a, OSC vs. SSC; c, OSC vs. NS; (right panel) representative images of pCREB immunostaining in distinct subfields of the dorsal hippocampus (top left) in the NS group (top right), SSC group (bottom left), and OSC group (bottom right). ANOVA, analysis of variance; BLA, basolateral amygdala; CA, cornu ammonis; CeA, central amygdala; CS, conditioned stimulus; DG, dentate gyrus; LA, lateral amygdala; NS, no-shock; OSC, optimal short conditioning; pCREB, phosphorylated cAMP response element binding; PVT, paraventricular nucleus of the thalamus; SSC, spaced short conditioning.

The computational model predicts that the increased overlap of PKA and ERK activation in the OSC protocol leads to greater LTP induction (inducer) in rat hippocampus. This increased overlap plausibly augments phosphorylation of CREB (29), a transcription factor that is required for synaptic plasticity and LTP [(22,61); see review by Kida (62)]. Therefore, we used immunohistochemistry to quantify levels of pCREB following fear conditioning in a no-shock control (NS) group and in the SSC and OSC groups (Figure 5A) in brain regions previously implicated in the acquisition of fear memory such as the amygdala, the paraventricular nucleus of the thalamus (PVT), and the dorsal hippocampus [(63,64); see review by Do-Monte *et al.* (65)]. During the fear acquisition session, rats that were exposed to SSC and OSC protocols exhibited higher freezing levels, which is indicative of successful fear acquisition, than the NS group, with the OSC group showing even higher freezing levels than the SSC group (Figure 5B), consistent with Figure 4.

Immunohistochemistry revealed high levels of pCREB expression in the SSC and OSC groups compared with the NS group across different brain regions including the lateral, basal, and central nuclei of the amygdala (Figure 5C), and the PVT (Figure 5D), suggesting that increased pCREB levels in the SSC and OSC groups are mediated by the CS-US pairing. However, we did not observe differences in pCREB levels between the OSC and SSC groups in the amygdala or PVT (Table 1), and pCREB levels were similar among the 3 groups in CA1 hippocampal subfields (Figure 5E). Interestingly, rats exposed to the OSC protocol exhibited increased pCREB expression in the dentate gyrus (DG) compared with the SSC and NS groups (Figure 5E). Given that increased pCREB in the DG has been associated with enhanced LTP in rodents (66), these data support the hypothesis that the augmentation in fear memory acquisition observed with the OSC protocol is mediated by higher levels of LTP induction in the rat hippocampus.

### The Optimal Protocol Also Enhanced Fear Extinction

**Figure 6:**
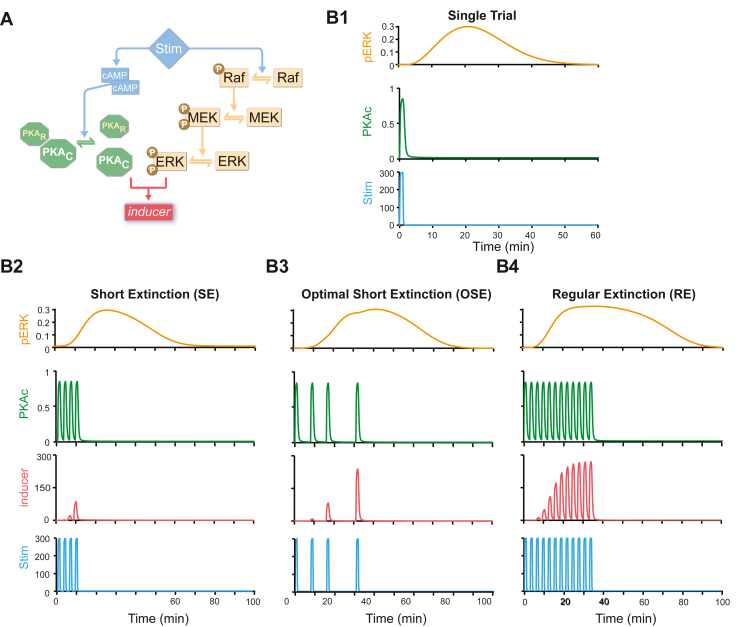
Prediction of enhanced protocol for fear extinction. **(A)** Schematic of the model. **(B1)** Simulated time courses of activated ERK (pERK, μM) and activated PKA (PKAc, μM) in response to 1 trial of Stim (μM). **(B2)** Simulated time courses of pERK, PKAc, and inducer in response to 4-trial protocol with regular intervals of 2.5 minutes (SE). **(B3)** Simulated time courses of pERK, PKAc, and inducer in response to 4-trial protocol with computationally designed intervals (OSE). **(B4)** Simulated time courses of pERK, PKAc, and inducer (nM) in response to 12-trial protocol with regular intervals of 2.5 minutes (RE). cAMP, cyclic adenosine monophosphate; ERK, extracellular signal-regulated kinase; MEK, mitogen-activated protein kinase; OSE, optimal short extinction; pERK, phosphorylated ERK; PKA, protein kinase A; PKAc, protein kinase A catalytic subunit; PKAr, protein kinase A regulatory subunit; RE, regular extinction; SE, short extinction; Stim, stimulus.

Extinction is new learning that temporarily inhibits the initial associative memory (67,68). Extinction also relies on PKA and ERK signaling to induce plasticity-related gene transcription (69, 70, 71). We predicted that the computationally designed protocol would also enhance the acquisition of extinction, thereby suppressing the original fear memory (Figure 6A–B1). We compared an OSE protocol comprising 4 trials with irregular ITIs of 8, 8, and 16 minutes (Figure 6B3) against an RE (Figure 6B4) protocol using 12 trials and ITIs of 150 seconds and an SE (Figure 6B2) protocol using 4 trials and the same ITIs of 150 seconds, which resemble previous protocols used for regular and short fear extinction in rats (72, 73, 74). In simulations, the OSE protocol triggered higher peak inducer than the SE protocol and was comparable to the RE protocol (Figure 6B1–3). Thus, we predicted that OSE would result in stronger extinction of fear memory than the standard SE.

**Figure 7:**
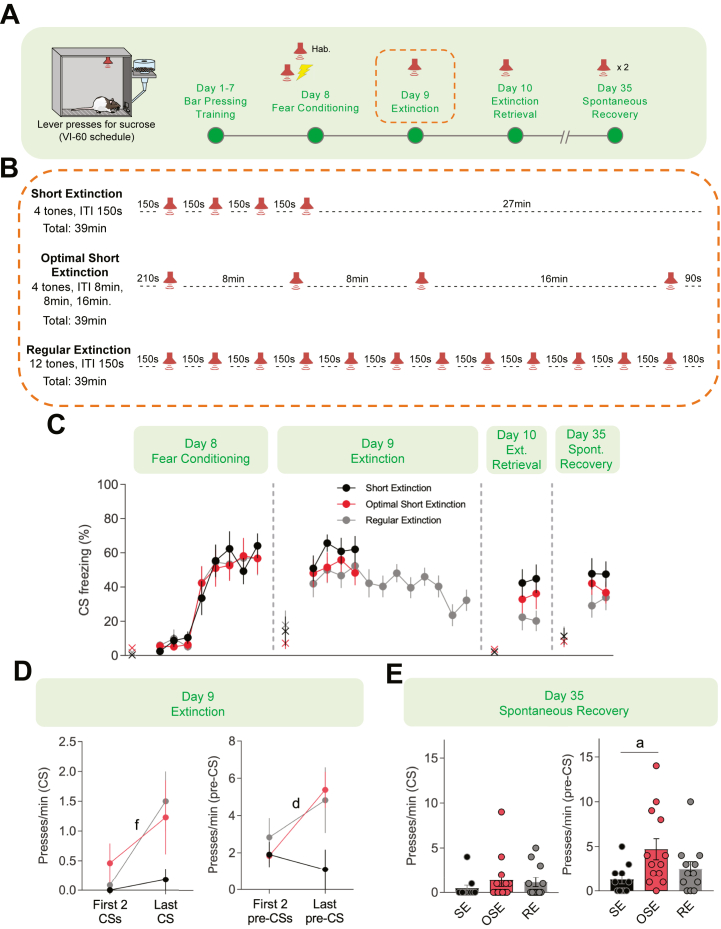
The computationally designed protocol partially enhanced fear extinction in rats. **(A)** Schematic of the enhanced fear extinction experimental procedures. Rats were trained to constantly press a lever to retrieve sucrose pellets in a VI60 schedule, where the average interval between each sucrose delivery is 60 seconds. **(B)** Schematics of the fear extinction protocols for SE (*n* = 11), OSE (*n* = 13), and RE (*n* = 12) groups, during which rats received multiple trials of a CS (3 kHz tone, 30 seconds). **(C)** Freezing levels during CS presentations of each group across the experiment. No significant difference between groups was found by two-way repeated measures analysis of variance. × denotes the pretone freezing levels. **(D)** Lever-press rates during the first 2 and the last CS presentation (left) or the 30 seconds before the first 2 and the last CS presentation (pre-CS period) of each group. OSE group shows significant increase of pre-CS lever presses comparing the last CS presentation with the average lever-press rate of the first 2 CS presentations. Paired Student’s *t* test. Letters f and d indicate tests with *p* < .05: f, RE, last CS vs. first 2 CS; d, OSE, last pre-CS vs. first 2 pre-CS. **(E)** The OSE group shows more lever presses during the pre-CS period than the SE group in spontaneous recovery test. No difference was found for the CS lever presses among the 3 groups (*H*_2_ = 2.102, *p* = .349). Kruskal-Wallis test followed by Dunn’s post hoc test. Letter a indicates pairwise comparison with *p* < .05: OSE vs. SE. CS, conditioned stimulus; ext., extinction; Hab., habituation; ITI, intertrial interval; OSE, optimal short extinction; RE, regular extinction; SE, short extinction; spont., spontaneous; VI, variable interval.

To test this prediction, we designed an experiment comparing extinction protocol efficacy using conditioned suppression of reward-seeking behavior (Figure 7A), which is more sensitive than freezing in fear extinction paradigms (75,76). Lever presses also help to maintain constant activity for reliable freezing measurement throughout the session (77). Because lever presses for reward are trained in a specific context, and extinction memory is context dependent (78,79), we only used one context in this experiment. Rats were initially trained to press a lever to receive sucrose pellets in a variable interval schedule of 60 seconds until they reached the same levels of lever pressing and locomotor activity (Supplemental Methods). On day 8, rats underwent a fear conditioning session which included 5 nonreinforced habituation tones followed by 7 CS-US pairings. On day 9, rats were preassigned to 3 experimental groups for extinction (Figure 7B) so that freezing levels, average speeds, and lever-press rates were similar during the fear conditioning session (Table 1). At the end of the extinction session, freezing levels reduced from ∼50% to ∼25% in the RE group, whereas the SE and OSE groups maintained the same levels of freezing throughout the 4 CS (Figure 7C, day 9). Similarly, the RE group showed a significant increase in lever presses during the CS presentations at the end of the session, whereas CS lever presses remained unaltered in the SE and OSE groups (Figure 7D, left). However, the OSE group showed a significant increase in lever presses during the 30-second periods preceding the CS (pre-CS) presentations (Figure 7D, right), suggesting enhanced within-session extinction of contextual fear memory (80).

On days 10 and 35, rats were returned to the same chamber to test the strength of fear extinction memory during extinction retrieval and spontaneous recovery tests, respectively. No significant difference in CS freezing or CS lever presses were observed among the 3 groups (Figure 7C, day 10 and 35). However, the OSE group showed increased pre-CS lever pressing during the spontaneous recovery test (Figure 7E, right) compared with the SE and RE groups. These results suggest that, although the tone-associated memory was similar among the groups, the OSE protocol enhanced contextual fear memory extinction, as evidenced by enhanced within-session extinction of conditioned suppression and reduced conditioned suppression 3 weeks postextinction training.

## Discussion

A computationally designed protocol that maximizes the interaction between PKA and ERK pathways enhances LTF and nonassociative learning in *Aplysia* (26). Here, we extended this strategy to associative learning in mammals by adapting the simplified mathematical model used with *Aplysia* to simulate the dynamics of PKA and ERK in rat hippocampus based on empirical data in the literature (33, 34, 35). Through a series of computational simulations and experimental validations, we discovered that an optimized training protocol, predicted to enhance the overlap of PKA and ERK activation, can facilitate acquisition and extinction of conditioned fear. Immunohistochemistry demonstrated that the augmented fear memory induced by the optimal protocol was associated with increased expression levels of pCREB in the DG subfield of the dorsal hippocampus. Our results demonstrate the power of a simplified model of intracellular signaling cascades in describing associative learning across species, attesting to the essential role of the interaction between PKA and ERK pathways in both nonassociative and associative learning.

Training protocols using spaced ITIs result in stronger memory acquisition than those using standard massed ITIs, a well-established phenomenon described as the spacing effect [see review by Smolen *et al.* (11)]. The spacing effect has been linked to the varying efficacy of massed and spaced protocols in triggering LTP/LTF via biochemical signaling, and a previous computational model suggests that the optimal ITI aligns with peak ERK activation (43,81,82). However, that model assumed fixed ITIs, which may not be ideal for synaptic plasticity and long-term memory. Our optimized training protocol, with irregular ITIs, induced stronger and more persistent fear memory in rats than a spaced protocol that had been predicted to be the most effective among protocols with equal ITIs. These results suggest that the spacing effect in mammals can be at least partially explained by enhanced overlap between the PKA and ERK pathways, which are critical for CREB activation (29). Our study also indicates that learning protocols with irregular ITIs may be more effective than those with equal ITIs. Although spaced protocols are known for facilitating fear memory acquisition, there is a lot of controversy when it comes to fear extinction. Whereas some studies have demonstrated that spaced intervals between the CS facilitate extinction memory and attenuate renewal and spontaneous recovery of fear (60,83, 84, 85), others have shown the opposite, impaired extinction memory and increased recovery of fear (60,84). Therefore, we selected a control group with massed trials to better control for our optimized protocol.

The enhanced performance of the optimal (i.e., the OSC) protocol appeared to be constrained by the intensity of the stimuli. The model predicted higher performance when weak stimuli were used but comparable performance with strong stimuli (Figure 4A). Drawing a line to distinguish weak and strong footshock intensities is not straightforward because the relationship between fear memory and footshock intensity is neither monotonic nor linear (86). Nevertheless, we observed clear differences in the efficacy of the optimal fear conditioning protocol when using footshocks of different intensities. When we compared the OSC protocol with a massed SC protocol using a standard footshock intensity (0.7 mA, Figure 2) commonly used in previous studies (28), we found only small differences in the conditioned responses (CS freezing) between the 2 protocols. However, when we used a lower footshock intensity (0.5 mA, Figure 4) to compare the OSC protocol with an SSC protocol, we found a significant increase in CS freezing in the OSC group during fear acquisition, retrieval, and spontaneous recovery tests. Considering the robustness of the spacing effect, it is possible that differences between the OSC and the massed SC groups at the higher footshock intensity were in part masked by a ceiling effect. Alternatively, previous studies with rats have demonstrated that the hippocampus is required for tone-evoked fear memory when a weak (0.4 mA) but not a strong (0.9 mA) footshock is used (87), which could also explain the differences we observed at the 2 intensities. Either way, our data suggest that our approach could be more beneficial for learning protocols that rely on relatively weak stimuli. Additional studies need to be conducted to understand the mechanism by which the intensity of stimuli governs the enhanced performance of protocols with irregular ITIs compared with protocols with fixed ITIs.

The association between the CS and US is primarily mediated by the lateral amygdala, where LTP induces enhanced CS responses [see review by Johansen *et al.*, (39) and Maren (88)]. Nevertheless, another important and distinguishable component of fear memory formation is the context in which the association has occurred. Interestingly, we only observed enhanced conditioned responses in the context in which fear conditioning occurred, suggesting that the memory facilitation effect is context dependent. Similarly, in the fear extinction experiment, enhancement by the optimal protocol was only observed during the pre-CS lever pressing, a more sensitive index of contextual fear memory during extinction and spontaneous recovery (75,76,80,89,90). This is consistent with the hippocampus’ role in encoding context during fear conditioning and extinction and in the time-dependent reappearance of fear after extinction (i.e., spontaneous recovery), on which our model is based [see reviews by Maren *et al.* (50) and Bouton *et al.* (91)]. Accordingly, immunohistochemical results showed greater pCREB expression in the OSC group specifically in the DG of the dorsal hippocampus, a region that has been implicated in the acquisition and extinction of contextual fear memory (92,93). Increasing CREB expression in DG neurons has been demonstrated to improve contextual fear acquisition (94), whereas inactivating DG neurons that overexpress CREB disrupts contextual fear retrieval (95).

Another hippocampal subregion that has been implicated in contextual fear is the CA1 (96). Although the OSC, SSC, and NC groups displayed similar levels of pCREB expression in CA1, the single posttraining time point (15 minutes) that we used does not suffice to rule out the possibility of distinct pCREB dynamics in CA1 compared with DG. CA1 pCREB peaks at 30 minutes and disappears at 90 minutes following fear conditioning (97,98). Additional experiments comparing distinct posttraining intervals may better reveal the dynamics of pCREB expression in different hippocampal subregions, as well as in other areas involved in fear acquisition (e.g., amygdala, PVT), which in the current study exhibited similar levels of pCREB expression in the OSC and SSC groups. The observation that the dynamics of ERK pathways differ across brain regions involved in fear memory, with peaks occurring at 20 minutes after a single stimulation in the hippocampus versus 60 minutes in the lateral amygdala (35,99,100), suggests that the model may be able to predict optimal protocols targeting specific components of fear memory based on the dynamics of intracellular signaling cascades in corresponding brain regions. Future studies will test this possibility by modeling the molecular cascades in the lateral amygdala and the medial prefrontal cortex to preferentially target the acquisition and extinction of CS-associated memories, respectively.

Our model is an obvious simplification of the mechanisms underlying fear conditioning and extinction. It does not include other molecular cascades that are critical for LTP and memory formation, such as CaMKII (calcium/calmodulin-dependent protein kinase II) or PKC (protein kinase C), due to the lack of empirical data for simulating their dynamics of activation (12,101,102). The model was constructed using empirical PKA and ERK dynamics from the literature, which are based on ex vivo analyses and have limited temporal resolution. Furthermore, the model assumes that the molecular mechanisms for fear memory acquisition are similar to those for extinction. Despite these limitations, the simplified model was sufficient to predict enhanced activation of the LTP-related transcription factor CREB in the DG and to facilitate associative learning in 3 different experiments. A more complex model that incorporates a wider range of intracellular and extracellular processes based on in vivo data would likely have enhanced predictive ability. Additional experiments should also investigate the memory enhancing effects of computationally designed training protocols in different types of associative memories, including discrimination learning and backwards conditioning. In addition, it will be important to test whether the effects observed with optimal protocols vary across subjects of different sexes and ages, as well as protocol efficacy in animal models of cognitive impairment. Taken together, our results suggest the possibility of using similar model-driven, noninvasive behavioral approaches in studies aimed at enhancing learning or restoring memory deficits in humans or improving extinction-based exposure therapies for anxiety disorders.

## Supplementary Material

Supplementary Data

Key Resources Table
