# Supplementary material for: Enhancing Associative Learning in Rats With a Computationally Designed Training Protocol
Source: Biol Psychiatry Glob Open Sci. 2023 Aug 1;4(1):165–81. doi: 10.1016/j.bpsgos.2023.07.006 (PMC10829654; doi:10.1016/j.bpsgos.2023.07.006)
Supplement: Supplementary Data [file mmc1.pdf]

SUPPLEMENTARY INFORMATION

Enhancing Associative Learning in Rats With a Computationally Designed Training Protocol

Zhang *et al.*

Supplementary Methods

Animals

All experimental procedures were approved by the Center for Laboratory Animal Medicine and Care of The University of Texas Health Science Center at Houston. National Institutes of Health guidelines for the care and use of laboratory animals were strictly followed in order to minimize any potential discomfort and suffering. A total of 120 male Long-Evans hooded adult rats (Charles Rivers Laboratories) 3-5 months of age and weighing 330-450 g were used. Rats were kept in a 12-hour light/12-hour dark cycle with food and water *ad libitum*. Experiments were conducted during the light phase. For the Optimal Extinction experiment, animals were maintained on a restricted diet of 18 g per day of standard laboratory rat chow to increase their motivation during the lever press training. Rats' weights were monitored weekly to make sure that all animals maintained their weight under food restriction.

Model Development

The mathematical model for the activation of kinase cascades critical for LTM was modified from a previous model of the signaling cascades for the induction of LTF (1). As

mentioned above, the induction of LTM for fear conditioning requires activation of multiple kinase cascades with different temporal dynamics. In the model, the cyclic AMP (cAMP)-PKA pathway is rapidly activated after training, whereas the Raf- MEK-ERK pathway is slowly activated, and both are required for the induction of LTM after fear conditioning and extinction (**Fig. 1A, Fig. 4A**).

PKA Pathway. The dynamics of cAMP activation upstream of PKA following training and the cAMP-dependent activation of PKA are described by Eqs. 1-4. Inactive PKA is a holoenzyme (PKA_{RC}, Eq. 2), consisting of regulatory (PKA_R, Eq. 3) and catalytic (PKA_C, Eq. 4) subunits. In response to training, represented by the variable *Stim* in Eq. 1, cAMP is activated (Eq. 1). Active cAMP binds to the regulatory subunit of PKA, leading to the release of free, active catalytic subunit (Fig. 1A) (Eqs. 2-4). '*Stim*' represents the neurotransmitters released by training that activate kinase cascades.

$$\frac{d[cAMP]}{dt} = \lambda \frac{[Stim]}{[Stim] + K_{Stim}} - k_{b,cAMP}[cAMP] \qquad \text{(Eq. 1)}$$

$$\frac{d[PKA_{RC}]}{dt} = k_{b,PKA}[PKA_C][PKA_R] - k_{f,PKA}[PKA_{RC}][cAMP]^2 \qquad \text{(Eq. 2)}$$

$$\frac{d[PKA_R]}{dt} = k_{f,PKA}[PKA_{RC}][cAMP]^2 - k_{b,PKA}[PKA_C][PKA_R] \qquad \text{(Eq. 3)}$$

$$\frac{d[PKA_C]}{dt} = k_{f,PKA}[PKA_{RC}][cAMP]^2 - k_{b,PKA}[PKA_C][PKA_R] \qquad \text{(Eq. 4)}$$

Parameter values are modified from (1) to activate PKA immediately after training, but with PKA activity quickly returning to the basal in ~5 min (Fig. 1B) (2, 3): λ = 14.6 µM/min, K_{STIM} = 85 µM, $k_{b,cAMP}$ = 4 min^{-1}, $k_{f,PKA}$ = 20 µM^{-2}min^{-1}, $k_{b,PKA}$ = 12 µM^{-1}min^{-1}

ERK Pathway. The activation of ERK by training '*Stim*' is via sequential activation of the upstream kinases Raf and MEK (Fig. 1A). Raf activates the MAP kinase kinase MEK, MEK in turn activates the MAP kinase ERK. The differential equations describing the activation of Raf, MEK, and ERK (Eqs. 5-12) are similar to those in (1). However, a discrete time delay in activation of Raf was removed from the phosphorylation of Raf (Eq. 5). Instead, based on empirical data (4, 5), parameters describing the activation of Raf were adjusted from (1) so that the ERK activation curve reached the peak around 20 min post-training (Fig. 1B).

$$\frac{d[Raf^{p}]}{dt} = k_{f,Raf}[Raf][Stim] - k_{b,Raf}[Raf^{p}]$$ (Eq. 5)

$$[Raf] = [Raf]_{total} - [Raf^{p}]$$ (Eq. 6)

$$\frac{d[MEK]}{dt} = \frac{k_{b,MEK}[MEK^{p}]}{[MEK^{p}] + K_{MEK,2}} - \frac{k_{f,MEK}[Raf^{p}][MEK]}{[MEK] + K_{MEK,1}}$$ (Eq. 7)

$$\frac{d[MEK^{pp}]}{dt} = \frac{k_{f,MEK}[Raf^{p}][MEK^{p}]}{[MEK^{p}] + K_{MEK,1}} - \frac{k_{b,MEK}[MEK^{pp}]}{[MEK^{pp}] + K_{MEK,2}}$$ (Eq. 8)

$$[MEK^p] = [MEK]_{total} - [MEK] - [MEK^{pp}] \quad \text{(Eq. 9)}$$

$$\frac{d[ERK]}{dt} = \frac{k_{b,ERK}[ERK^p]}{[ERK^p] + K_{ERK,2}} - \frac{k_{f,ERK}[MEK^{pp}][ERK]}{[ERK] + K_{ERK,1}} \quad \text{(Eq. 10)}$$

$$\frac{d[ERK^{pp}]}{dt} = \frac{k_{f,ERK}[MEK^{pp}][ERK^p]}{[ERK^p] + K_{ERK,1}} - \frac{k_{b,ERK}[ERK^{pp}]}{[ERK^{pp}] + K_{ERK,2}} \quad \text{(Eq. 11)}$$

$$[ERK^p] = [ERK]_{total} - [ERK] - [ERK^{pp}] \quad \text{(Eq. 12)}$$

Parameter values: $k_{f,Raf}$ = 0.001 μM^{-1}min^{-1}, $k_{b,Raf}$ = 0.05 min^{-1}, $[Raf]_{total}$ = 0.5 μM,

$k_{f,MEK}$ = 0.41 min^{-1}, $k_{b,MEK}$ = 0.04 μM/min, $K_{MEK,1}$ = 0.20 μM,

$K_{MEK,2}$ = 0.19 μM, $[MEK]_{total}$ = 0.5 μM, $k_{f,ERK}$ = 0.41 min^{-1},

$k_{b,ERK}$ = 0.12 μM/min, $K_{ERK,1}$ = 0.19 μM,

$K_{ERK,2}$ = 0.21 μM, $[ERK]_{total}$ = 0.5 μM.

As in (1), a variable '*inducer*' was used to quantify the overlap of activation between PKA and ERK, which together regulate the gene expression necessary for the induction of LTM.

$$inducer = k_{inducer}\,[PKA_C]\,[ERK^{pp}] \quad \text{(Eq. 13)}$$

where $k_{inducer}$ = 1 μM^{-1}.

To determine which protocols could more effectively activate *inducer,* four-trial protocols with three ITIs, each ranging from 2–20 min in steps of 2 min, were simulated. Combining these permutations yielded 1,000 protocols. For each protocol, the maximal (peak) overlap between PKA and ERK was quantified as the peak level of *inducer* produced. Also, the value of *Stim* was varied from 100 to 300 µM to represent weak and strong trainings and to test the robustness of protocols. Based on the maximal overlap, a protocol with ITIs of 8, 8, and 16 min was selected for optimal conditioning and extinction.

Numerical methods. Fourth-order Runge-Kutta integration was used for integration of all differential equations with a time step of 3 s. Further time step reduction did not lead to significant improvement in accuracy. The steady-state basal levels of variables were determined after at least one simulated day, prior to any manipulations. The model was programmed in XPPAUT (http://www.math.pitt.edu/~bard/xpp/xpp.html) (6) and simulated on Dell Precision T1700 microcomputers.

Behavioral Tasks

Optimal Conditioning

Apparatuses

Two distinct chambers (context A and context B) positioned inside sound attenuating boxes were used during the Optimal Conditioning experiments. Context A consisted of a small operant chamber (34 cm high x 25 cm wide x 23 cm deep, 200 lux, Med Associates, see schematic drawing in Fig. 2A top) with one of the two aluminum walls covered by black adhesive paper and two transparent acrylic walls, and a metal grid floor beneath which a microcentrifuge tube (Eppendorf) containing 50 µl of 10% amyl acetate (Sigma-

Aldrich) was positioned. Context B consisted of a larger acrylic operant chamber (40 cm high x 50 cm wide x 26 cm deep, 20 lux, Med Associates, see schematic drawing in Fig. 2A bottom) with one of its walls covered by a black and white striped paper, and a floor made of a white acrylic board beneath which a microcentrifuge tube (Eppendorf) containing 50 µl of deionized water was positioned.

Procedures

On day 0, rats were placed in context A for a 20-min familiarization session. Next, rats were pre-assigned to three experimental groups based on their baseline freezing and locomotor activity during the familiarization session: Regular Conditioning (RC), Short Conditioning (SC), and Optimal Short Conditioning (OSC). On day 1, rats were placed into context A and exposed to one nonreinforced habituation tone (3 kHz, 75 dB, 30 s) followed by distinct fear conditioning protocols (44 min duration). The RC group received eight presentations of a conditioned stimulus (CS, 3 kHz tone, 75 dB, 30 s) that co-terminated with an unconditioned stimulus (US, footshock, 0.7 mA, 0.5 s), with fixed ITIs of 270 s. The SC group received four CS-US pairings with the same ITIs of 270 s, and remained in the chamber until the end of the session. The OSC group received the computationally designed optimal protocol of four CS-US pairings with ITIs of 8, 8, and 16 min. On day 2, rats were placed in context B and given two CS presentations (ITI of 150 s, 7 min duration) in the absence of US to test the retrieval of tone-associated fear memory in a novel context. On day 3, rats were returned to context A for an extinction training session where they received twelve CS presentations (ITIs of 150 s, 37 min duration). The first four CSs were compared to the last four CSs to assess extinction

learning within the same session. On day 4 and 5, rats were placed back in context A and B, respectively, and exposed to an extinction retrieval session (similar to day 2). On day 29 and 30, rats were placed back in context A and B, respectively, and exposed to a spontaneous recovery session (similar to day 2). On day 1 and day 2, rats in the same group were trained simultaneously in four chambers with the group order counterbalanced to avoid interference from different protocols in neighboring chambers. On the following days, rats were simultaneously tested in four chambers regardless of the group assignment. Each rat was tested in the exact same chamber across the days. Footshocks, tones, intertrial intervals, and session duration were controlled by an automated video tracking system (ANY-maze, Stoelting), which also quantified the percentage of time freezing, distance traveled, average speed and maximum speed. All rats passed the criteria of 20% of freezing during at least one CS of the fear conditioning session and the first two CSs of the extinction session.

Optimal vs. Spaced Short Conditioning

Apparatus and Procedures

The same apparatus and contexts described above were used. Following familiarization on Day 0, rats were pre-assigned to two experimental groups based on their baseline freezing and locomotor activity during the familiarization session: Optimal Short Conditioning (OSC) and Spaced Short Conditioning (SSC). The same procedures described above were used here, except that: *i)* the footshock intensity was reduced from 0.7 mA to 0.5 mA to decrease the possibility of ceiling effects; *ii)* the SSC group received four CS-US pairings with ITIs of 11 min 10 s. The same automated video tracking system

(ANY-maze, Stoelting) described above was used for protocol control and behavioral quantification. One rat that failed the criteria of 20% of freezing during at least one CS of the fear conditioning session and the first two CSs of the extinction session was excluded from the analyses. For immunohistochemistry quantification of pCREB, an additional No-Shock (NS) control group was exposed to the same number of CS presentations and ITIs as the SSC group, but without the US (footshock).

Optimal Extinction

Lever-press training

Rats were placed in an acrylic/aluminum operant chamber (34 cm high x 25 cm wide x 23 cm deep, Med Associates, see schematic drawing in Fig. 4A) and trained to press a lever for sucrose on a fixed ratio of one pellet for each press. Next, animals were trained in a variable interval schedule of reinforcement that was gradually reduced across the days (one pellet every 15 s, 30 s, or 60 s) until they reached a minimum criterion of 10 presses/min after 7 days of training. All sessions lasted 30 min and were performed on consecutive days. Sucrose pellet delivery, variable intervals, and session duration were controlled by an automated video tracking system (ANY-maze, Stoelting).

Apparatus and Procedure

On day 8, rats were placed into the same chamber where they had previously undergone lever presses training. Animals were exposed to five nonreinforced habituation tones (3 kHZ, 75 dB, 30 s duration) followed by seven CS-US pairings (ITIs of 150 s, 37 min duration). The footshock intensity was increased from 0.7 mA to 1.0-1.2 mA to result in

stronger fear acquisition and consequently higher freezing levels during the extinction training session in the next day. Rats were pre-assigned to three experimental groups based on their freezing and lever presses during the fear conditioning session: Regular Extinction (RE), Short Extinction (SE), and Optimal Short Extinction (OSE). On day 9, rats were returned to the same chamber for a fear extinction session (39 min duration). The RE group received twelve CSs with ITIs of 150 s; the SE group received four CSs with ITIs of 150 s and remained in the chamber until the end of the session; and the OSE group received the computationally designed optimal protocol of four CSs with ITIs of 8, 8, and 16 min. On day 10 and 35, rats were placed into the same chamber and received two CSs with an ITI of 150 s to test the strength of fear extinction memory during extinction retrieval and spontaneous recovery tests, respectively. The same automated video tracking system (ANY-maze, Stoelting) described above was used for protocol control and behavioral quantification. Two rats that never reached the criterium of 20% of freezing during at least one CS of the fear conditioning session and the first two CSs of the extinction session were excluded from the analyses.

Immunohistochemistry

Rats were perfused with 200 ml of 0.1 M potassium phosphate buffer (KPBS) followed by 500 ml of 4% paraformaldehyde (PFA) in 0.1 M sodium phosphate buffer 15 min after day 1 of conditioning. Brains were removed from the skull, transferred to a 20% sucrose solution in PFA for 24 h, and stored in a 30% sucrose solution in PFA for another 24 h. Next, coronal brain sections (40 µm thick) were cut with a cryostat (CM1860, Leica) and stored in an antifreeze solution overnight. Sections were blocked in 20% normal goat

serum, 0.3% Triton X-100, and 3% hydrogen peroxide in KPBS at room temperature for 1 h. Brain sections were then incubated with anti-pCREB serum raised in rabbit (1:1000; EMD Millipore 06-519) overnight at room temperature. After sections were washed with KPBS five times, they were incubated with biotinylated goat anti-rabbit IgG antibody (1:200, Vector Labs, BA-1000-1.5) for 2 h, followed by incubation in ABC kit (1:50, Vector Labs, PK-6100) for 90 min and DAB-Ni solution (Vector Labs, SK-4100) for 25 min. Sections were washed with KPBS and mounted on Superfrost Plus slides. After three days, sections were serially dehydrated with alcohol (Epredia; 50 and 70% for 5min each, 95 and 100% for 10 min each) and defatted with Citrisolv (Decon Labs) for 30 min. Sections were then cover-slipped with mounting medium (Permount, Fisher Scientific) before being analyzed microscopically.

Microscopy and image analysis

Images were generated with a Nikon microscope (Eclipse NiE Motorized Upright Microscope) equipped with a fluorescent lamp (X-Cite, 120 LED) and a digital camera (Andor Zyla 4.2 PLUS sCMOS). Counts of pCREB-positive cells were performed at 20 x magnification. Cell detection and quantification for the immunohistochemistry experiment were performed automatically using QuPath (7) by an experimenter blind to the experimental groups. pCREB-positive cell detection was performed using QuPath's optical density sum with a requested pixel size of 0.5 μm. Nucleus parameters included a background radius of 6 μm, median filter radius of 0 μm, sigma of 1.5 μm, minimum area of 2 μm^2, and maximum area of 250 μm^2. Cell expansion was set to 0 μm. Intensity parameters included a threshold from 0.1-0.15 and maximum background intensity of 5,

and intensity threshold parameters were threshold 1 of 0.2, threshold 2 of 0.4, and threshold 3 of 0.6. pCREB-positive cells were automatically counted with a circular region of interest (0.228 mm^2) in each subfield of rat dorsal hippocampus including CA1, CA2, CA3, and DG, as well as in the lateral nucleus of the amygdala (LA), the basal nucleus of the amygdala (BA), the central nucleus of the amygdala (CeA) and the paraventricular nucleus of the thalamus (PVT) according to the rat brain atlas (8), and averaged for both hemispheres at 2-3 different antero-posterior levels from -2.4 mm to -4.2 mm from bregma. These brain regions were selected because they have been previously implicated in the acquisition of conditioned fear responses (9, 10). The density of pCREB-positive cells (cells per mm^2) was calculated by dividing the number of pCREB-positive cells by the area of the circular region of interest.

Quantification and statistical analysis

Rats were recorded with digital video cameras (Logitech C920) and all behavioral indices were measured using an automated video-tracking system (ANY-maze). Lever presses per minute were calculated by measuring the number of presses during the 30 s cue (or 30 s pre-cue) multiplied by two. All graphics and numerical values reported in the figures are presented as mean ± standard error of the mean (SEM). Grubbs' tests (11) were used to identify outliers ($p < 0.05$) in each experiment and one rat in Fig. 2 was removed after identification by the test. Shapiro-Wilk tests were performed to determine whether the data have a normal distribution. F tests before pair-wise comparison and Brown-Forsythe tests before multiple group comparison were performed to test if groups have the same variance. Statistical significance for parametric distributions was determined with paired

Student's t-test, Welch's t-test, one-way analysis of variance (ANOVA) or two-way repeated-measures ANOVA followed by Tukey or Holm-Sidak post-hoc comparisons, whereas Kruskal-Wallis test followed by Dunn's post-hoc test was used for non-parametric distributions (Software Prism 7), as indicated for each experiment. Sample size was based on estimations from previous literature and experience.

Supplementary References

1. Zhang Y, Liu RY, Heberton GA, Smolen P, Baxter DA, Cleary LJ, et al. (2011): Computational design of enhanced learning protocols. *Nat Neurosci*. 15:294-297.
2. Roberson ED, Sweatt JD (1996): Transient activation of cyclic AMP-dependent protein kinase during hippocampal long-term potentiation. *The Journal of biological chemistry*. 271:30436-30441.
3. Vázquez SI, Vázquez A, Peña de Ortiz S (2000): Different hippocampal activity profiles for PKA and PKC in spatial discrimination learning. *Behavioral Neuroscience*. 114:1109-1118.
4. Ajay SM, Bhalla US (2004): A role for ERKII in synaptic pattern selectivity on the time-scale of minutes. *Eur J Neurosci*. 20:2671-2680.
5. Wang Y, Zhu G, Briz V, Hsu YT, Bi X, Baudry M (2014): A molecular brake controls the magnitude of long-term potentiation. *Nature communications*. 5:3051.
6. Ermentrout B (2002): *Simulating, analyzing, and animating dynamical systems : a guide to XPPAUT for researchers and students*. Philadelphia: Society for Industrial and Applied Mathematics.
7. Bankhead P, Loughrey MB, Fernandez JA, Dombrowski Y, McArt DG, Dunne PD, et al. (2017): QuPath: Open source software for digital pathology image analysis. *Scientific reports*. 7:16878.
8. Paxinos G, Watson C (2014): *Paxino's and Watson's The rat brain in stereotaxic coordinates*. Seventh edition. ed. Amsterdam ; Boston: Elsevier/AP, Academic Press is an imprint of Elsevier.
9. Herry C, Johansen JP (2014): Encoding of fear learning and memory in distributed neuronal circuits. *Nat Neurosci*. 17:1644-1654.
10. Do-Monte FH, Quinones-Laracuente K, Quirk GJ (2015): A temporal shift in the circuits mediating retrieval of fear memory. *Nature*. 519:460-463.
11. Grubbs FE (1969): Procedures for Detecting Outlying Observations in Samples. *Technometrics*. 11:1-21.
